# Evaluation of the Antioxidative, Antibacterial, and Anti-Inflammatory Effects of the *Aloe* Fermentation Supernatant Containing *Lactobacillus plantarum* HM218749.1

**DOI:** 10.1155/2016/2945650

**Published:** 2016-07-17

**Authors:** Meixiu Jiang, Kan Deng, Chunling Jiang, Mingui Fu, Chunlan Guo, Xiaolei Wang, Xin Wang, Fanjing Meng, Shaoguo Yang, Keyu Deng, Tingtao Chen, Hongbo Xin

**Affiliations:** ^1^Institute of Translational Medicine, Nanchang University, Nanchang, Jiangxi 330031, China; ^2^Jiangxi Cancer Hospital, Nanchang 330045, China; ^3^Department of Basic Medical Science, School of Medicine, University of Missouri Kansas City, Kansas City, MO 64108, USA; ^4^College of Forestry, Jiangxi Agriculture University, Nanchang 330045, China

## Abstract

Little work is done to develop* Aloe vera* (AV) using probiotics. To explore the potential benefits, the antioxidant effects and the antibacterial effects on foodborne pathogens of* Aloe* fermentation supernatant were evaluated in vitro. Our results indicated that the* Aloe* fermentation supernatant fermented by* Lactobacillus plantarum* HM218749.1 had very strong scavenging capacities of the DPPH (86%), O_2_^•−^ (85%), ^•^OH (76%), and Fe^2+^ chelation (82%) and reducing powers (242.5 mg/L), and the inhibition zones for* Salmonella typhimurium*,* Salmonella enteritidis*,* Shigella flexneri*,* Escherichia coli*,* Listeria monocytogenes*,* S. dysenteriae* 301,* Staphylococcus aureus* Cowan1, and* Propionibacterium acnes* were 16, 15, 19, 20, 21, 20, and 27 mm. Moreover, the low concentration of* Aloe* fermentation supernatant had significantly reduced the production of IL-1*β*, TNF-*α*, and IL-6 in both mRNA and protein levels (*P* < 0.01). Therefore, the* Aloe* fermentation supernatant can be used as functional beverage or cosmetic ingredients to guard human intestinal health, delaying senescence, and prevent chronic diseases.

## 1. Introduction

*Aloe vera* (AV) is a cactus-like plant which has been used widely in herbal medicines for millennia [1]. The compounds in fleshy leaves, for example, aloe emodin, aloin, aloesin, saponins, terpenoids, and polysaccharides, possess the wound healing, anti-inflammatory, immunity, antidiabetic, antioxidant, laxative, antibacterial, antifungal, antiviral, and antitumor effects [2–6]. Now, AV has also been widely used as cosmetic-moisturizers, tooth pastes, food flavoring compounds, and preservative in the pharmaceutical and food fields [2]. However, little work is done to exploit the AV using probiotics.

Probiotics are defined as “live microorganisms which when administered in adequate amounts, confer a health benefit on the host.” Previous studies indicated that probiotics conferred miscellaneous health benefits include an improvement in lactose intolerance, an increase in natural resistance to infectious disease in the gastrointestinal tract, the suppression of cancer, an antidiabetic agent, and a decrease in serum cholesterol levels [7–9]. In recent decades, probiotics are the hotspot for microbiologist and nutritionists [10, 11], and the health awareness and busy lifestyle have created a strong and dynamic probiotics market which offers good prospects for consumers [12].

Among the probiotics,* Lactobacillus plantarum* is pointed out as an industrially important microorganism that can be found and isolated from dairy products, fermented foods (sauerkraut, sourdough, sausages, cheeses, wines, olives, and pickled vegetables), environments (cow-dung, silage, and sewage), human mouth, intestinal tract, and stools [13, 14], and this strain had been widely used for the development of functional foods and potential oral vaccines [13]. In our previous work [15],* L. plantarum* HM218749.1 was isolated from human intestinal, which could grow better at 37°C in an anaerobic environment and could resist harsh conditions of gastrointestinal tract (pH 2.5 and 0.30% bile salt). Moreover, this strain could strongly adhere to HT-29 cells and reduce the adhesion of* Shigella dysenteriae *2457,* Staphylococcus aureus* Cowan1,* Enterobacter sakazakii* 45401, and* Escherichia coli* 44102 to HT-29 cells, and its long-term residence in mice intestine increased the number of lactobacilli and decreased the number of enterococci [15].

In the present study, the tested probiotics (e.g.,* Bifidobacterium longum* ATCC15708,* L. plantarum* HM218749,* L. rhamnosus* GG, and* Streptococcus thermophilus* G1) grew well in AV substrate and conferred AV sound antioxidant and antibacterial effects, and the* L. plantarum* HM218749.1 was finally chosen for its popularity in food fields and perfect beneficial effects verified in our previous studies, and the cell experiment was carried out to further verify the anti-inflammatory effects of the* Aloe* fermentation supernatant.

## 2. Materials and Methods

### 2.1. Preparation of AV Extract and* Aloe* Fermentation Supernatant

The AV leaves were freshly harvested by cutting from the bottom and were washed thoroughly. The edges were removed and the leaves were peeled to obtain the slimy and transparent gel contents. The gel was then mashed using a pulper, which was then centrifuged at 4000 ×g for 10 min to obtain the AV extract [1].

The probiotic* Bifidobacterium longum* ATCC15708 (isolated from the milk),* L. plantarum *HM218749 (isolated from human intestine),* L. rhamnosus* GG (isolated from the milk), and* Streptococcus thermophilus* G1 (isolated from the milk) were cultured in de Man Rogosa Sharpe (MRS) medium (for* Lactobacilli* and* Bifidobacterium*) and brain/heart infusion (BHI) medium (for* Streptococcus thermophilus* G1) for 24 h, and 10^8^ cfu/mL of the strains was used as an inoculum to be added to* Aloe vera*,* Aloe vera* + 5% glucose, or* Aloe vera* + 5% glucose + 5% skimmed milk for preparing the* Aloe* fermentation supernatant, which was incubated for 15 to 18 h at 37°C or 42°C. Finally, the* Aloe* fermentation supernatant and AV were freeze-dried using vacuum freeze drier under with the following conditions: freeze drying at 0.2 mbar vacuum, −40°C for 48 h. The lyophilized powder was stored at −20°C for further use.

### 2.2. Cultivable Bacterial Counts

Bacterial counts were enumerated as previously reported [16]. Diluted aliquots were replica-plated onto BHI agar and MRS agar and then incubated anaerobically in the anaerobic system (full of 85% N_2_, 5% H_2_, and 10% CO_2_) at 37°C or 42°C for 24~36 h followed by counting colonies from plates showing 25–250 colonies [17].

### 2.3. Antioxidative and Antibacterial Activity of AV Extract and* Aloe* Fermentation Supernatant

For preparation of the* Aloe* fermentation supernatant, 10% of the probiotics were added to AV extract and fermented for 15 to 18 h at 37°C, and then the* Aloe* fermentation cultures were centrifuged at 4000 ×g for 10 min, and the pellets in the bottom of the centrifuge tubes (containing insoluble materials or/and probiotics) were deemed as the residual substance, and the clear water insoluble substance was deemed as supernatant, and the AV extract was used as a control. Then, the clearance of the DPPH, O_2_^•−^, ^•^OH, Fe^2+^ chelation, and oxygen-reduction activity were measured just as in [18]. For antimicrobial activity, overnight (12 h) cultures of the pathogenic microorganisms including* Salmonella typhimurium* ATCC 13311,* S. enteritidis* ATCC13076,* S. flexneri* ATCC 12022,* E. coli* 44102,* Listeria monocytogenes* ATCC 19111,* S. dysenteriae* 301,* S. aureus* Cowan1, and* Propionibacterium acnes* ATCC 11827 were spread on the surface of the LB agar plates, and the culture supernatant (200 *μ*L) was loaded into an Oxford cup (outer diameter 7.8 ± 0.1 mm, inner diameter 6.0 ± 0.1 mm, and height 10.0 ± 0.1 mm), which was placed on the surface of the agar. The size of the inhibition zone was measured until a formation of clear zone around the Oxford cup. The experiment was carried out in duplicate [17].

### 2.4. Cell Viability Assay

RAW 264.7 cells (passages 8–20) were maintained in DMEM supplemented with 10% FBS and antibiotics (100 U/mL of penicillin and 100 *μ*g/mL of streptomycin) at 37°C in a 5% CO_2_ incubator. Emodin (Wuhan qi Boster Biological Technology co. Ltd.), polysaccharide (Wuhan qi Boster Biological Technology co. Ltd.),* Aloe vera* (lyophilized powder), and* Aloe vera* +* L. plantarum* (lyophilized powder) were dissolved in phosphate-buffered saline (PBS) solution and their effect on cell viability of RAW 264.7 cells was determined by MTT assay [19].

RAW 264.7 cells were plated at a density of 10000 per well in 100 *μ*L of complete culture medium and treated with designed concentrations of emodin (0, 1, 2.5, 5, and 10 *μ*g/mL), polysaccharide (0, 2.5, 5, 15, 30, and 50 *μ*g/mL),* Aloe vera* (0, 5, 15, 30, 50, 100, and 200 *μ*g/mL), and* Aloe vera* +* L. plantarum* (0, 5, 15, 30, 50, 100, and 200 *μ*g/mL) in 96-well microtiter plates for 36 h at 37°C in a humidified incubator. Control cells treated with PBS served as the vehicle group. Each concentration of drugs was repeated in 5 wells. After incubation for specified times, MTT reagent (20 *μ*L, 5 mg/mL) was added to each well and incubated for 4 h. After careful removal of the MTT solution, 150 *μ*L of DMSO was added to each well. The absorbance was recorded on a microplate reader at the wavelength of 570 nm.

### 2.5. Anti-Inflammatory Evaluation of AV Extract and* Aloe* Fermentation Supernatant Using RT-PCR and ELISA

RAW 264.7 cells were seeded into 24-well plates (5 × 10^4^ cells per well) for 24 h and were then washed with PBS. Cells were treated with/without lipopolysaccharide (LPS, 1 *μ*g/mL each), emodin (2.5 *μ*g/mL and 10 *μ*g/mL), polysaccharide (2.5 *μ*g/mL and 15 *μ*g/mL),* Aloe vera* (5 *μ*g/mL and 50 *μ*g/mL), and* Aloe vera* +* L. plantarum* (5 *μ*g/mL and 50 *μ*g/mL) for 24 h, and then the cells and supernatants were harvested for Q-PCR and ELISA analysis, respectively.

For the evaluation of cytokine mRNA expression levels, total RNAs from the RAW 264.7 cells were prepared by adding TRIzol reagent (Gibco BRL, Grand Island, NY, USA) according to the manufacturer's protocol, and 2.4 *μ*g of total RNA from each group was reverse transcribed to cDNA using a commercially available kit (Applied Biosystems). Quantitative real-time PCR was performed with 7900HT fast real-time PCR system (ABI, Foster City, CA) using 2 × SYBR Green master mix (Bio-Rad). Forty cycles were conducted as follows: 95°C for 30 s and 60°C for 30 s, preceded by 1 min at 95°C for polymerase activation with the following primers (Q-PCR IL-1*β*: sense primer 5′-GTGTCTTTCCCGTGGACCTTC′-3′, antisense primer 5′-TCATCTCGGAGCCTGTAGTGC-3′: Q-PCR TNF-*α*: sense primer 5′-GTGGAACTGGCAGAAGAGGCA, antisense primer 5′-AGAGGGAGGCCATTTGGGAAC-3′ Q-PCR IL-6: sense primer 5′-GGAAATCGTGGAAATGAG-3′, antisense primer 5′-GCTTAGGCATAACGCACT-3′ Q-PCR GAPDH: sense primer 5′-CTCGTGGAGTCTACTGGTGT-3′, antisense primer 5′-GTCATCATACTTGGCAGGTT-3′).

The products of IL-1*β*, TNF-*α*, and IL-6 in cell supernatants were determined using the ELISA kit for IL-1*β* (eBioscience), TNF-*α* (eBioscience), and IL-6 (eBioscience).

### 2.6. Data Analysis

Data are reported as means ± SD, and results were analyzed using SPSS 13.0 software (SPSS Inc., Chicago, IL, USA) by means of independent one-way ANOVA tests in each sampling point. The differences among the three groups were assessed by means of the least significant difference (LSD) multiple comparison test (*P* < 0.05 or 0.01).

## 3. Results

### 3.1. Exploration of the Fermentation Conditions of Probiotics

**Figure 1:**
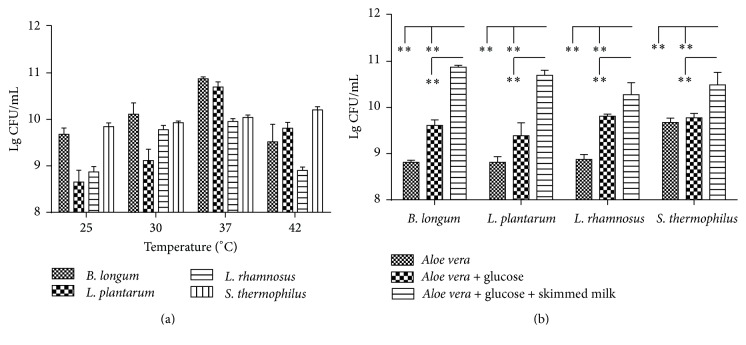
The suitable temperature (a) and fermentation substrates (b) for* B. longum*,* L. plantarum*,* L. rhamnosus* GG, and* S. thermophilus*. The significant analysis was done between* Aloe vera*,* Aloe vera* + glucose, and* Aloe vera* + glucose + skimmed milk. Data are shown as the mean ± SD. ^*∗∗*^ *P* < 0.01.

To determine the optimum growth temperature of the selected probiotics, the same initial inoculums of* B. longum* (MRS medium),* L. plantarum* (MRS medium),* L. rhamnosus* LGG (MRS medium), and* S. thermophilus* (BHI medium) were inoculated in the corresponding media and cultured at 25°C, 30°C, 37°C, and 42°C, and the results showed that the optimum temperature for* B. longum*,* L. plantarum*, and* L. rhamnosus* LGG was 37°C, and* S. thermophilus* received its maximum yield at 42°C (Figure 1(a)).

To further investigate the effects of different additives on the growth of probiotics, glucose and skimmed milk were added in the AV extract, and the results indicated that all the tested strains grew well in AV extract with the initial pH 6.0 (about 9 Lg CFU/mL, Figure 1(b)). It seemed that the addition of 5% glucose and 5% glucose + 5% skimmed milk had significantly enhanced the biomass of* B. longum*,* L. plantarum*, and* L. rhamnosus* GG (*a* < 0.01), and the growth promoting effect of the 5% glucose + 5% skimmed milk was much better than 5% glucose (*b* < 0.01) (Figure 1(b)).

### 3.2. Antioxidative and Antibacterial Activity of* Aloe* Fermentation Supernatant

To evaluate the additive of the probiotics on the perfect antioxidant and antibacterial activities of AV [9, 20–22], the AV + 5% glucose, which gives us more choices for the future products, was chosen for our next study.

Compared with the AV, the addition of probiotics made little change on O_2_^•−^ clearance, Fe^2+^ chelation, and reduction activity, while the* L. plantarum* HM218749.1 had significantly (*P* < 0.01) enhanced the DPPH clearance and ^•^OH clearance (Table 1). Considering the sound probiotic properties of the* L. plantarum* HM218749.1 [15], this strain was finally chosen for the antibacterial evaluation.

**Figure 2:**
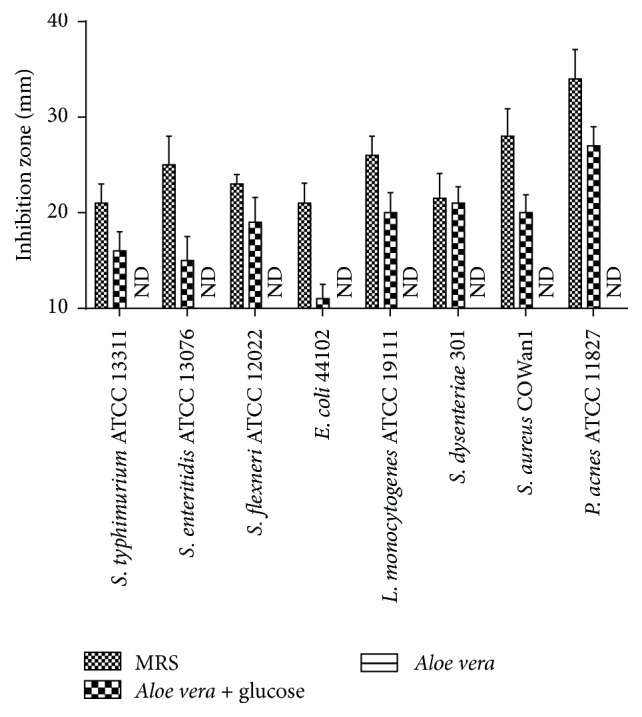
Antibacterial activities of* L. plantarum* against selected foodborne pathogens cultured in MRS,* Aloe vera* + glucose, and* Aloe vera*. Data are shown as the mean ± SD.

Interestingly, no antimicrobial effect was observed using AV extract and* Aloe* fermentation supernatant (fermented by* L. plantarum* HM218749.1 without additional glucose), while the addition of 5% glucose conferred the* Aloe* fermentation supernatant a perfect inhibition to all the tested pathogens, for example,* S. typhimurium* ATCC 13311 (inhibition zone diameter: 16 mm),* S. enteritidis* ATCC13076 (IZD: 15 mm),* S. flexneri* ATCC 12022 (IZD: 19 mm),* E. coli* 44102 (IZD: 11 mm),* L. monocytogenes* ATCC 19111 (IZD: 20 mm),* S. dysenteriae* 301 (IZD: 21 mm), and* S. aureus* COWAN1 (IZD: 20 mm) (Figure 2). Most of all, the* Aloe* fermentation supernatant possessed the best antimicrobial effect on* P. acnes* ATCC 11827 (IZD: 27 mm), which leads to an accumulation of the skins natural oils, bacteria, and acne vulgaris [23].

### 3.3. The Anti-Inflammatory Activity of the* Aloe* Fermentation Supernatant on Proinflammatory Cytokines

During inflammation, excess levels of cytokines (IL-1*β*, IL-6, and TNF-*α*) lead to the damaging of cells and tissues and the activation of macrophages in inflammation-associated diseases (e.g., rheumatoid arthritis and chronic hepatitis) [24, 25].

**Figure 3:**
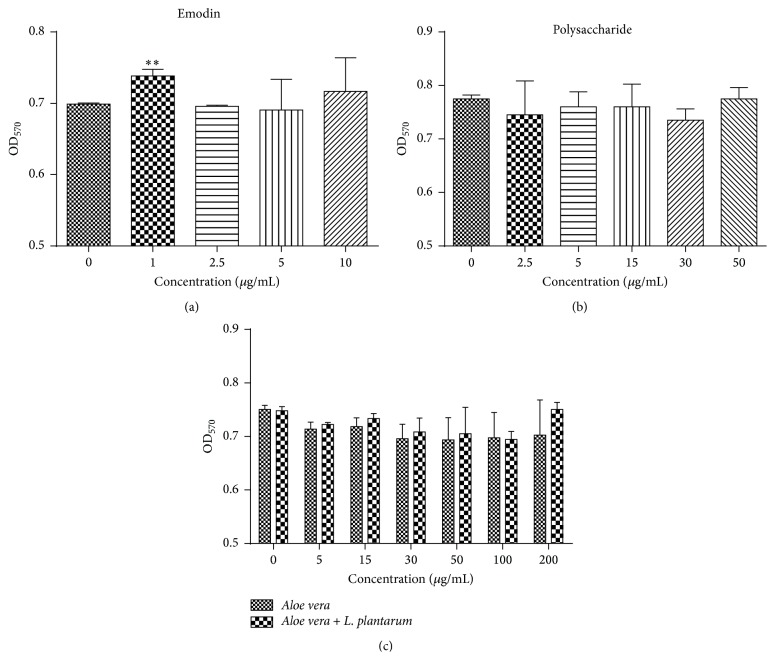
Different concentration of emodin (a), polysaccharide (b),* Aloe vera* and* Aloe vera* +* L. plantarum* (c) on the cell viability of RAW 264.7 cells using MTT method. Data are shown as the mean ± SD. ^*∗∗*^ *P* < 0.01.

To check the functional components of AV and* Aloe* fermentation supernatant on the production of IL-1*β*, IL-6, and TNF-*α*, different concentrations of emodin (0, 1, 2.5, 5, and 10 *μ*g/mL), polysaccharide (0, 2.5, 5, 15, 30, and 50 *μ*g/mL), AV extract (0, 5, 15, 30, 50, 100, and 200 *μ*g/mL), and* Aloe* fermentation supernatant (0, 5, 15, 30, 50, 100, and 200 *μ*g/mL) were cocultured with RAW 264.7 cells to test their toxicity on RAW 264.7 cells, and the results indicated that no obvious toxicity was observed for all the tested materials, and the low concentration of emodin (1 *μ*g/mL) showed promoting effects on the growth of RAW 264.7 cells (Figure 3).

Then, low and high doses of the above drugs were determined to evaluate the anti-inflammatory effects of* Aloe* fermentation supernatant according to the previous studies [1, 20, 22, 26, 27]. The real-time PCR results indicated that all the tested emodin, polysaccharide, AV extract, and* Aloe* fermentation supernatant had significantly reduced the inflammatory factor of IL-1*β*, IL-6, and TNF-*α* in gene level, except for the polysaccharide at 15 *μ*g/mL. In protein level, our ELISA results showed that both the AV extract and* Aloe* fermentation supernatant presented significantly inhibition on IL-1*β*, IL-6, and TNF-*α* production, and it seemed that the inhibition effect of AV extract was better than* Aloe* fermentation supernatant. However, little inhibition effect of emodin and polysaccharide on IL-1*β* and polysaccharide on IL-6 was observed (Figure 5).

## 4. Discussion

As we all know, the single target drug had been widely used in various human diseases but often failed to cure the metabolic diseases [28]. Thus, the whole-body systems biology was proposed and regarded the human and symbiotic microorganisms as a whole “superorganism” [28, 29], which led a more and more reorganization by the scientific community to the Chinese herb extracts and the human intestinal probiotics.

Previously studies showed that both the AV [2, 22] and probiotics [25, 30, 31] had sound antioxidant and anti-inflammatory effects, so we guessed that the combination of the AV and probiotics should possess better antioxidative, antibacterial, and anti-inflammatory effects. First, we optimized the optimum growth temperature of the selected probiotics and found that the probiotics could grew well in AV extract without any additives (Figure 1). However, no antimicrobial effects of AV and* Aloe* fermentation were observed which was inconsistent with the previous studies [2, 32] (Figure 2). We guessed that the lack of glucose, the substrate for probiotics to produce antimicrobial production of lactic acid and acetic acid, was the cause of the antimicrobial failure for* Aloe* fermentation; therefore we added 5% glucose to AV extract and found that the added glucose not only significantly increased the probiotics growth (Figure 1) but also conferred the* Aloe* fermentation supernatant sound antibacterial effects on all the tested pathogens (Figure 2).

After a series of evaluations, the* L. plantarum* HM218749.1 was chosen and this strain conferred the* Aloe* fermentation supernatant strong scavenging capacities of the DPPH (86%), O_2_^•−^ (85%), ^•^OH (76%), Fe^2+^ chelation (82%), and reducing powers (242.5 mg/L) (Table 1). Moreover, the* Aloe* fermentation fermented by* L. plantarum* HM218749.1 could inhibit all the growth of tested pathogens, especially for the* P. acnes* ATCC 11827 (IZD: 27 mm). Therefore, the sound inhibition of the growth of* P. acnes* ATCC 11827 (causative bacteria for acne vulgaris) and antioxidant activity made the* Aloe* fermentation supernatant a potential ingredient for cosmetic.

As we know, excess levels of IL-1*β*, IL-6, and TNF-*α* induced by activated inflammatory cells (e.g., eosinophils, macrophages, mononuclear phagocytes, neutrophils) will lead to the damage of cells and tissues, which eventually cause the inflammation-associated diseases; for example, rheumatoid arthritis, chronic hepatitis, and macrophages play an important role in regulating several immunopathological conditions and inducing overexpression of the proinflammatory mediators of the inflammatory process [24, 25]. So the RAW 264.7 cells were used in the present study to evaluate the anti-inflammatory effect of* Aloe* fermentation supernatant fermented by* L. plantarum* HM218749.1.

**Figure 4:**
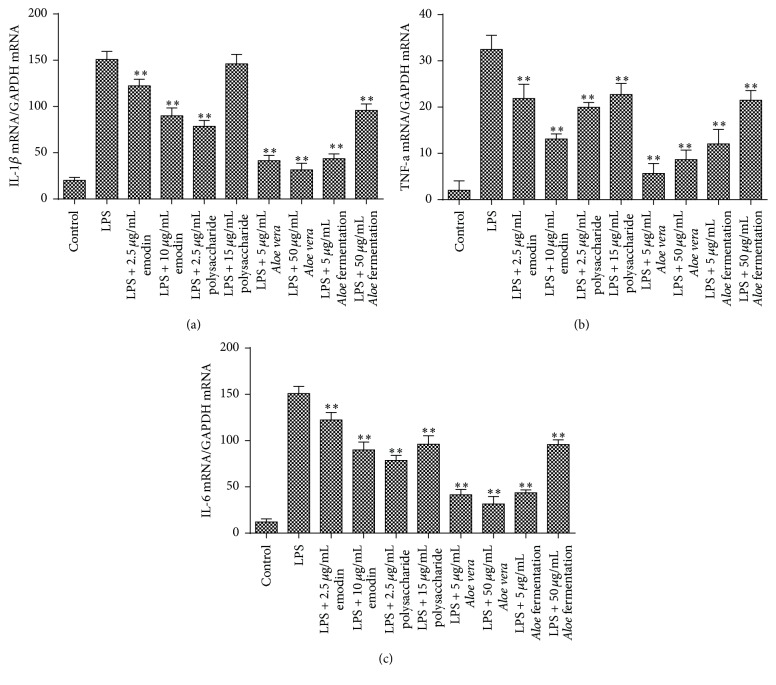
Effect of the different concentrations of emodin, polysaccharide,* Aloe vera*, and fermentation broth on the expression of inflammatory genes (IL-1*β*, TNF-*α*, and IL-6) in RAW 264.7 cells. Data are shown as the mean ± SD. ^*∗∗*^ *P* < 0.01.

**Figure 5:**
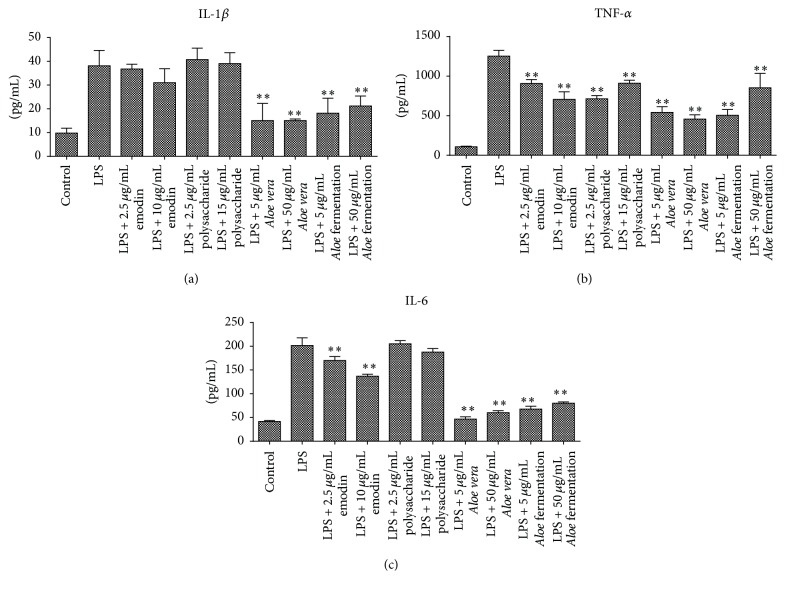
Effect of the different concentrations of emodin, polysaccharide,* Aloe vera*, and fermentation broth on the protein yields of IL-1*β*, TNF-*α*, and IL-6 in RAW 264.7 cells. Data are shown as the mean ± SD. ^*∗∗*^ *P* < 0.01.

The results indicated that both the emodin (anticancer activity) [33] and the polysaccharide (immunity enhancement and reduction of oxidative injury) showed weaker anti-inflammatory effects compared to AV extract and* Aloe* fermentation supernatant in mRNA level and protein level (Figures 4 and 5). Interestingly, though the* Aloe* fermentation supernatant possessed a strong antioxidant effect (Figure 2), its anti-inflammatory effect was inferior to the AV extract in both gene level and protein level. Maybe, some anti-inflammatory ingredients in AV extract were consumed by* L. plantarum* HM218749.1, and the metabolites of this strain might contribute to the inflammatory response of RAW 264.7 cells. Moreover, in vivo work is needed because of the shortcomings of cell model; for example, it could not model the interaction between intestinal cells and microbiota. As potential probiotics, the* L. plantarum* HM218749.1 could produce bacteriocins offering advantages in colonization and competition in the gastrointestinal tract [34] and endowed the AV extract a strong antioxidative effect and antimicrobial effect on pathogens (especially for* P. acnes*). In addition, the* L. plantarum* HM218749.1 could remove the bitter taste of AV, and our human results (*n* = 15) indicated that the* Aloe* fermentation supernatant had shortened the acne cure time and had a perfect whitening effect (data unshown).

In conclusion, the* Aloe* fermentation supernatant's sound antimicrobial effect and anti-inflammatory effect, together with its intestinal health promoting effect when* L. plantarum* HM218749.1 enters host intestines, indicate its potential use as functional foods or cosmetics to guard human intestinal health, delaying senescence and preventing chronic diseases.

## Figures and Tables

**Table 1:** The antioxidant activity of the *Aloe vera*, *Aloe vera* + *B. longum*, *Aloe vera* + *L. plantarum*, *Aloe vera* + *L. rhamnosus* GG, and *Aloe vera* + *S. thermophilus*.

Group	DPPH clearance (%)	O_2_^•−^ clearance (%)	^•^OH clearance (%)	Fe^2+^ chelation (%)	Reducing powers (mg/L)
*Aloe vera*	63.46 ± 4.13	92.34 ± 7.12	68.23 ± 2.23	83.51 ± 4.78	0.244 ± 0.032
*Aloe vera* + *B. longum*	56.73 ± 6.12	94.28 ± 5.48	74.29 ± 1.54^*∗∗*^	82.34 ± 5.41	0.246 ± 0.09
*Aloe vera* + *L. plantarum*	85.23 ± 4.23^*∗∗*^	93.13 ± 4.23	76.23 ± 6.72^*∗∗*^	82.87 ± 6.23	0.242 ± 0.13
*Aloe vera* + *L. rhamnosus*	55.23 ± 3.48	92.12 ± 5.33	59.87 ± 5.81^*∗∗*^	87.23 ± 6.12	0.239 ± 0.08
*Aloe vera* + *S. thermophilus*	82.13 ± 2.31^*∗∗*^	93.19 ± 7.12	61.52 ± 6.78^*∗∗*^	83.23 ± 3.34	0.251 ± 0.11

Note: data are shown as the mean ± SD. ^*∗∗*^ *P* < 0.01.
